# Strain dyssynchrony index determined by three-dimensional speckle area tracking can predict response to cardiac resynchronization therapy

**DOI:** 10.1186/1476-7120-9-11

**Published:** 2011-04-05

**Authors:** Kazuhiro Tatsumi, Hidekazu Tanaka, Takayuki Tsuji, Akihiro Kaneko, Keiko Ryo, Kohei Yamawaki, Alaa MS Omar, Yuko Fukuda, Kazuko Norisada, Kensuke Matsumoto, Tetsuari Onishi, Akihiro Yoshida, Hiroya Kawai, Ken-ichi Hirata

**Affiliations:** 1 Division of Cardiovascular Medicine, Department of Internal Medicine, Kobe University Graduate School of Medicine, Kobe, Japan

**Keywords:** heart failure, pacemakers, echocardiography

## Abstract

****Background**:**

We have previously reported strain dyssynchrony index assessed by two-dimensional speckle tracking strain, and a marker of both dyssynchrony and residual myocardial contractility, can predict response to cardiac resynchronization therapy (CRT). A newly developed three-dimensional (3-D) speckle tracking system can quantify endocardial area change ratio (area strain), which coupled with the factors of both longitudinal and circumferential strain, from all 16 standard left ventricular (LV) segments using complete 3-D pyramidal datasets. Our objective was to test the hypothesis that strain dyssynchrony index using area tracking (ASDI) can quantify dyssynchrony and predict response to CRT.

****Methods**:**

We studied 14 heart failure patients with ejection fraction of 27 ± 7% (all≤35%) and QRS duration of 172 ± 30 ms (all≥120 ms) who underwent CRT. Echocardiography was performed before and 6-month after CRT. ASDI was calculated as the average difference between peak and end-systolic area strain of LV endocardium obtained from 3-D speckle tracking imaging using 16 segments. Conventional dyssynchrony measures were assessed by interventricular mechanical delay, Yu Index, and two-dimensional radial dyssynchrony by speckle-tracking strain. Response was defined as a ≥15% decrease in LV end-systolic volume 6-month after CRT.

**Results:**

ASDI ≥ 3.8% was the best predictor of response to CRT with a sensitivity of 78%, specificity of 100% and area under the curve (AUC) of 0.93 (p < 0.001). Two-dimensional radial dyssynchrony determined by speckle-tracking strain was also predictive of response to CRT with an AUC of 0.82 (p < 0.005). Interestingly, ASDI ≥ 3.8% was associated with the highest incidence of echocardiographic improvement after CRT with a response rate of 100% (7/7), and baseline ASDI correlated with reduction of LV end-systolic volume following CRT (r = 0.80, p < 0.001).

****Conclusions**:**

ASDI can predict responders and LV reverse remodeling following CRT. This novel index using the 3-D speckle tracking system, which shows circumferential and longitudinal LV dyssynchrony and residual endocardial contractility, may thus have clinical significance for CRT patients.

## Background

Cardiac resynchronization therapy (CRT) is an established therapy for advanced heart failure (HF) patients with wide QRS duration[1-8]. However, it is well known from randomized clinical trials and single-center studies that the proportion of patients considered clinical or echocardiographic non-responders has remained at roughly one-third. The reason why so many patients selected according to standard clinical criteria are non-responders to CRT could be associated with lack of mechanical dyssynchrony, myocardial scar tissue, left ventricular (LV) lead position associated with the site of latest mechanical activation, and insufficient atrioventricular (AV) and/or venoventricular (VV) optimization. Accordingly, it would be useful to assess these factors in combination that may influence response to CRT. Lim et al[9] reported that the strain delay index, determined by two-dimensional (2-D) longitudinal speckle tracking strain, is coupled with the factors of both left ventricular (LV) dyssynchrony and residual myocardial contractility[9]. Moreover, we recently reported that our relatively simple version of the strain dyssynchrony index obtained with 2-D speckle strains can also predict response to CRT, and that combining assessment of radial, circumferential, and longitudinal strain dyssynchrony index can further improve the prediction of responders[10].

A newly developed three-dimensional (3-D) speckle tracking system using complete 3-D pyramidal datasets can quantify LV dyssynchrony[11,12]. More recently, this novel 3-D speckle tracking system has been found to be able to quantify the endocardial area change ratio (area strain), when coupled with the factors of both longitudinal and circumferential strain from all 16 standard LV segments. The objective of our study was to test the hypothesis that strain dyssynchrony index using area tracking (ASDI) can qualify dyssynchrony and residual myocardial function, and predict response to CRT and LV reverse remodeling. This report presents both the results of our investigation and a discussion of the clinical implications.

## Methods

### Study Population

The study included 15 consecutive HF patients with New York Heart Association functional class III or IV, ejection fraction ≤ 35%, and QRS duration ≥ 120 msec, who underwent CRT. One of these patients was excluded from all subsequent analyses because of poor echocardiographic image quality, so that eventually 14 patients were enrolled in this study (Table 1). Their mean age was 70 ± 9 years, ejection fraction was 27 ± 7%, and QRS duration was 172 ± 30 msec. Five patients (36%) had ischemic cardiomyopathy, defined as the presence of ≥75% stenosis of at least one major epicardial coronary artery and/or prior coronary revascularization. Ten patients (71%) were diagnosed with sinus rhythm and there was no patient with atrial fibrillation. In addition, four of the patients (29%) had previously undergone implantation of a permanent right ventricular (RV) pacing device at least one year before enrollment and featured predominantly RV pacing, which was defined as ≥ 90% paced when the device was interrogated at the time of enrollment. Whenever tolerated, all patients were on optimal pharmacological therapy. Written informed consent was obtained from all patients.

**Table 1:** Baseline characteristics of patients and their response to CRT

Variable	All Patients (14)	Responders (9)	Non-Responders (5)	p value
Age ( years )	70 ± 9	70 ± 8	71 ± 12	NS
Male/female	12/2	8/1	4/1	NS
NYHA functional class III/IV	12/2	8/1	4/1	NS
QRS duration (ms)	172 ± 30	178 ± 32	163 ± 26	NS
SR/AF/paced	10/0/4	6/0/3	4/0/1	NS
End-diastolic volume (ml)	175 ± 72	194 ± 82	140 ± 29	NS
End-systolic volume (ml)	132 ± 67	152 ± 76	94 ± 20	NS
Ejection fraction (%)	27 ± 7	23 ± 6	32 ± 3	NS
Heart failure etiology				
Ischemic	5 (36%)	2 (22%)	3 (60%)	NS
Non-ischemic	9 (64%)	7 (78%)	2 (40%)	NS
Medication				
ACEI/ARB	11 (92%)	7 (89%)	4 (100%)	NS
β-blockers	11 (92%)	7 (89%)	4 (100%)	NS
Diuretics	10 (83%)	6 (75%)	4 (100%)	NS
Area strain dyssynchrony index (%)	4.1 ± 1.4	4.8 ± 1.1	2.8 ± 0.7	< 0.005
Conventional dyssynchrony measurements (ms)				
Yu index	49 ± 17	48 ± 19	51 ± 15	NS
IVMD	46 ± 28	53 ± 31	34 ± 15	NS
Radial dyssynchrony determined by Speckle tracking strain	233 ± 129	277 ± 114	153 ± 126	NS

ACEI, angiotensin converting enzyme inhibitor; AF, atrial fibrillation; ARB, angiotensin receptor blocker; IVMD, Interventricular mechanical delay; NS, not significance; NYHA, New York Heart Association; SR, sinus rhythm.

### Echocardiographic Examination

All echocardiographic studies were performed with a 2.5-MHz 3-D matrix array transducer and 3-MHz sector transducer using a commercially available echocardiography system (Aplio Artida, Toshiba Medical Systems, Tochigi, Japan). Patients were studied before and 6 ± 1 months after CRT. We acquired digital LV 3-D volume data from the apical view with 4- or 6-beat electrocardiogram-gated acquisition. Routine digital grayscale 2-D and tissue Doppler cine loops were obtained, including mid-LV short axis views at the level of the papillary muscle and standard apical views (4-chamber, 2-chamber, and long-axis). Sector width was optimized to allow for complete myocardial visualization while maximizing the frame rate. Mean volume rates were 22 ± 3 volumes/s in the apical views for gray scale imaging used for 3-D speckle tracking analysis, 45 ± 4 frames/s in the short axis view for gray scale imaging used for 2-D speckle tracking analysis, and 44 ± 3 frames/s in the apical axis view for tissue Doppler imaging used for tissue Doppler velocity analysis. LV end-diastolic volume, end-systolic volume (ESV), and ejection fraction were obtained with the modified biplane Simpson's method[13]. A response to CRT was defined as a reverse remodeling detected by a relative reduction of ≥ 15% in ESV at the 6-month follow-up after CRT.

### Strain Dyssynchrony Index using Area Tracking (ASDI)

**Figure 1:**
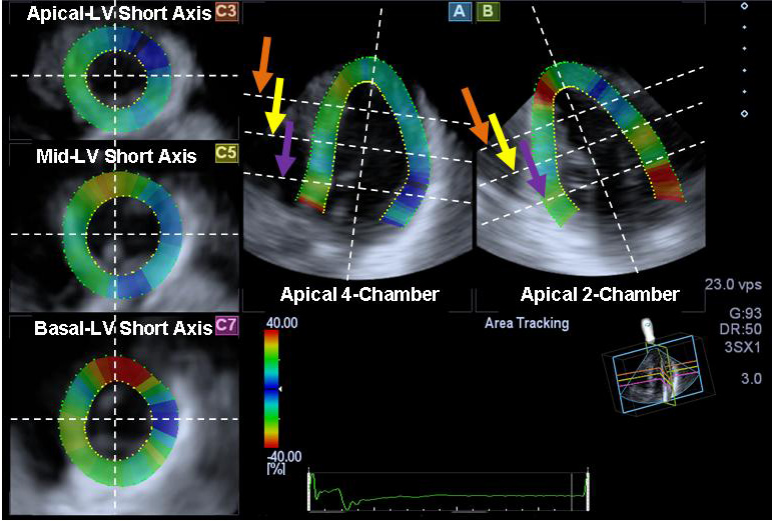
**Example of images generated from a pyramidal three-dimensional dataset: (A) apical 4-chamber view, (B) 2-chamber view, (C3) apical left ventricular (LV) short-axis view (orange arrow), (C5) mid-LV short-axis view (yellow arrow), and (C7) basal LV short-axis view (purple arrow)**.

**Figure 2:**
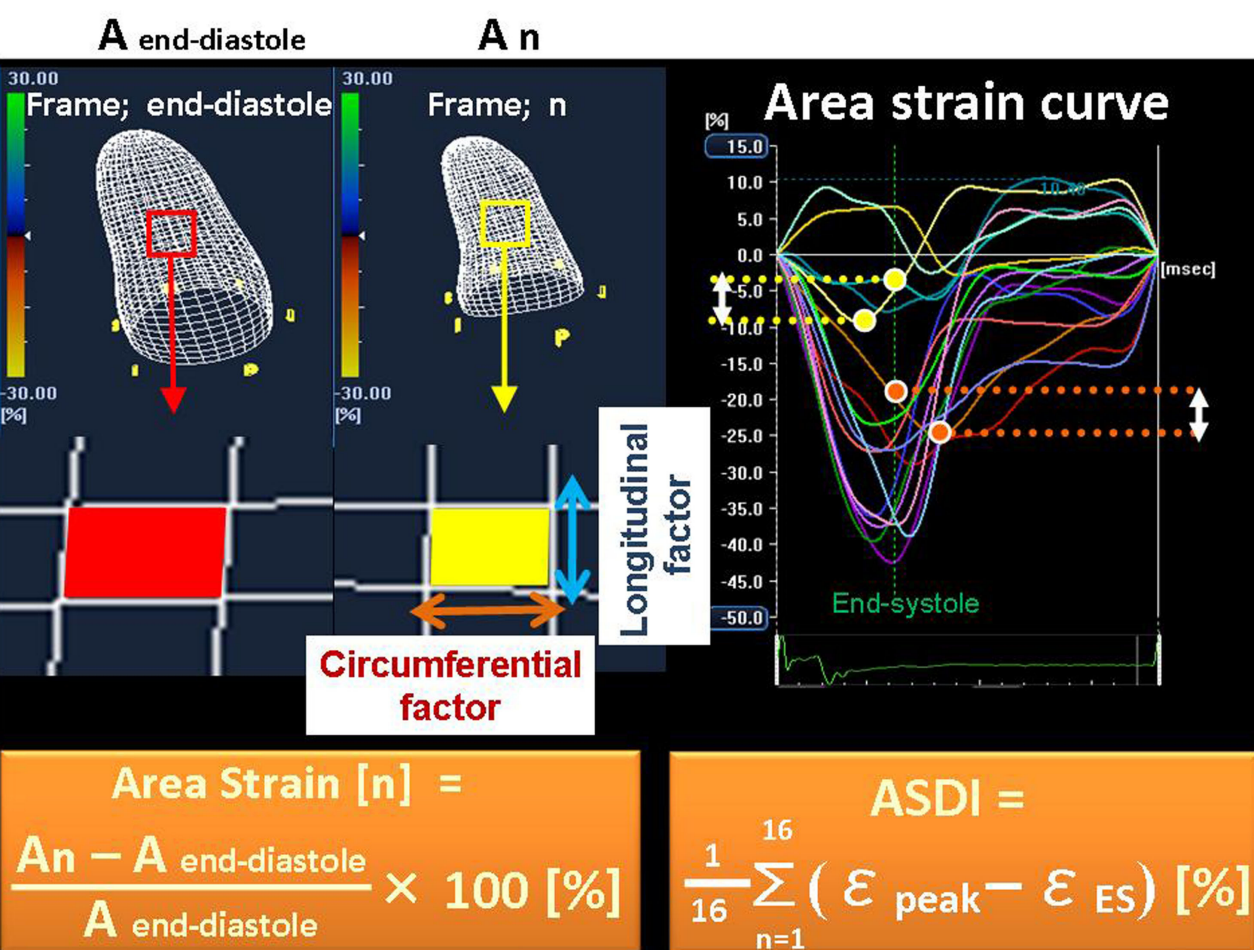
**The left panel shows the concept of three-dimensional (3-D) area strain**. Area strain expresses how the change in a regional area of the endocardium in a phase vs. that in the diastolic phase. Area strain is combined with both 3-D longitudinal and circumferential factors. The right panel shows an example of the strain dyssynchrony index using area tracking (ASDI). ASDI was calculated as the wasted energy per segment due to dyssynchrony and was determined by using the average difference between peak strain (ε-peak) and end-systolic strain (ε-ES) from 16 segments.

**Figure 3:**
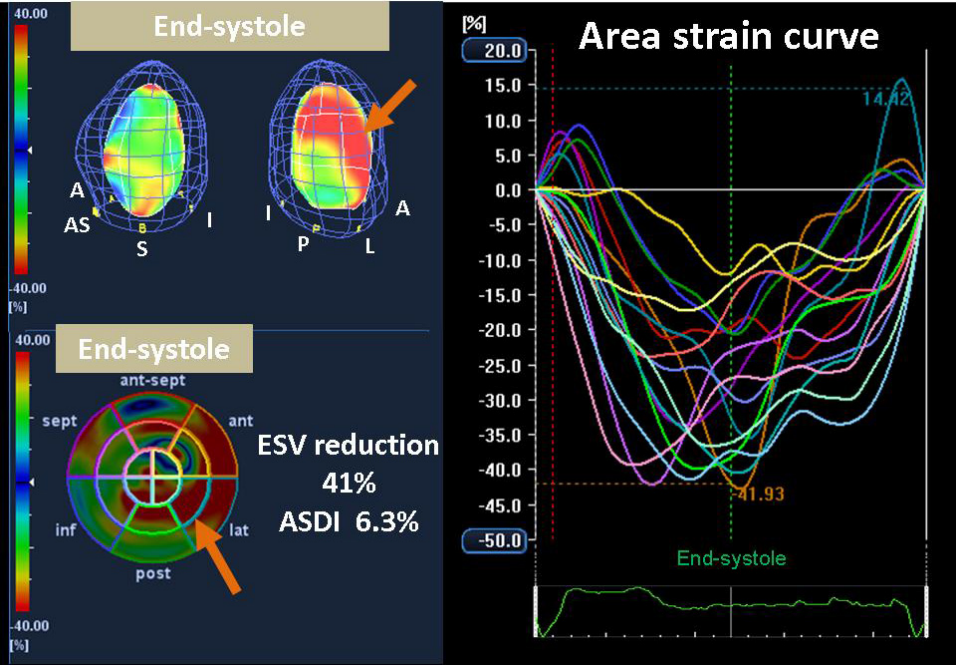
**Color-coded 3-D LV display *(top left) *and bull's-eye plot image *(bottom left) *and corresponding area strain curves from 16 LV sites *(right) *for a responder, demonstrating dyssynchronous strain curves represented by heterogeneous coloring at end-systole, with delayed peak strain in lateral segments (orange arrow)**. A and ant = anterior; AS and ant-sept = anterior-septum; I and inf = inferior; L and lat = lateral; P and post = posterior; S and sept = septal.

Our relatively simple version of the strain dyssynchrony index with 2-D speckle tracking strains represents the average of the wasted energy due to LV dyssynchrony as previously described in detail[10]. In the current study, the strain dyssynchrony index was calculated by means of area tracking. Briefly, ASDI analysis was performed with the aid of 3-D speckle area tracking strain using complete 3-D pyramidal datasets. First, a region of interest was traced on the endocardial cavity with a point and click approach. A second larger concentric circle was then automatically generated and manually adjusted near the epicardium (Figure 1). The 3-D area strain curve of the endocardium was calculated automatically (Figure 2 and 3). ASDI was then calculated as the average difference between peak and end-systolic area strain derived from 16-segment (Figure 2). The peak of the Q wave on the electrocardiogram was automatically used as the reference time point for end-diastole. The timing of minimum LV volume, determined by means of 3-D speckle tracking echocardiography, was used as the reference time point for end-systole. The wasted energy per segment of the endocardium as a result of dyssynchrony was expressed as the difference between peak strain (ε-peak) and end-systolic strain (ε-ES) (Figure 2). This difference increases with an increase in the degree of dyssynchrony, because this increase leads to a decrease in ε-ES. ASDI was then calculated as the average of the absolute difference between ε-peak and ε-ES derived from 16 segments. Three cardiac cycles were recorded and averaged for each measurement. If a segment showed a positive area strain during the entire cardiac cycle, the difference between ε-peak and ε-ES was assumed to be 0.

### Conventional Dyssynchrony Measurements

#### Interventricular dyssynchrony analysis

Routine pulsed-Doppler was used to determine interventricular dyssynchrony as previously described[14,15]. Interventricular mechanical delay (IVMD) was determined as the time difference between the onset of RV ejection and the onset of LV ejection. An IVMD of ≥ 40 ms was considered to constitute significant dyssynchrony [14,15].

#### Longitudinal tissue Doppler dyssynchrony analysis

Longitudinal dyssynchrony was determined by using tissue Doppler cine loops from three consecutive beats which were obtained in three standard apical views as previously described in detail[14,16]. Regions of interest (6 × 8 mm) were placed on the basal and mid-LV segments for each of the three standard views for a 12-site analysis of time to peak velocity. Longitudinal tissue Doppler dyssynchrony was determined as the standard deviation of time to peak systolic velocities from the onset of the QRS complex at the 12 sites (Yu Index)[14,16]. A longitudinal tissue Doppler dyssynchrony of ≥ 32 ms was considered to be significant[14,16].

#### 2-D Speckle Tracking Dyssynchrony Analysis

For dyssynchrony analysis by 2-D speckle tracking radial strain, routine gray scale mid-LV short-axis images were used as previously described in detail[14,17]. Briefly, an end-diastolic circular region of interest was traced on the endocardial cavity with a point-and click approach. A second larger concentric circle was then automatically generated and manually adjusted near the epicardium. The software automatically divided the mid-LV short-axis and apical images into six standard segments and provided the radial speckle tracking strain curves throughout the cardiac cycle. 2-D radial dyssynchrony detected by speckle tracking strain was defined as the time difference between the anteroseptal and posterior wall segmental peak strains[14,15,17-20]. A 2-D radial dyssynchrony of ≥ 130 ms was considered to constitute significant dyssynchrony[14,15,17-20].

#### Biventricular Pacemaker Implantation

A biventricular pacing system was implanted with an RV apical lead and an LV lead through the coronary sinus in all patients. The LV lead was placed in the lateral vein of seven patients, the posterolateral vein of four patients, and the anterolateral vein of three patients. Device implantation was successful for all patients without any major complications. The AV interval was adjusted for optimal diastolic filling by Doppler echocardiographic assessment of mitral inflow[21], and the VV interval was adjusted by Doppler echocardiographic assessment of LV out flow 8 ± 1 days after implantation[22,23].

### Reproducibility of Measurements

The interobserver and intraobserver reproducibility of the measurements of ASDI for randomly selected 10 patients was evaluated by two independent observers. To assess the interobserver reproducibility, measurements for all patients were analyzed by the second observer blinded to the values obtained by the first observer. For assessment of the intraobserver reproducibility, measurements of all patients were analyzed on 2 consecutive days by an observer blinded to the results of the previous measurements. The intraclass correlation coefficient was used to evaluate interobserver and intraobserver reproducibility.

### Statistical Analysis

Continuous variables were expressed as mean ± SD. The paired *t *test was used to compare group data obtained before and 6 months after CRT, and the unpaired Student's *t *test for comparison of group data for responders and non-responders. The diagnostic performance of LV dyssynchrony indexes for predicting response to CRT was evaluated by means of receiver operating characteristic curve analysis. Univariate linear correlation analysis was used for a comparison of baseline ASDI and reduction in ESV after CRT. For all tests, p value < 0.05 was considered statistically significant. All the analyses were performed with commercially available software (SPSS version 15.0, SPSS Inc., Chicago, IL, USA).

## Results

### Patient Characteristics

The baseline clinical and echocardiographic characteristics of the 14 patients are summarized in Table 1. Overall findings showed that CRT reduced LV end-diastolic volume and LVESV (from 175 ± 72 to 153 ± 68 ml, p < 0.005 and from 132 ± 67 to 98 ± 53 ml, p < 0.001, respectively) and increased LV ejection fraction (from 27 ± 7 to 38 ± 9%, p < 0.001). Response to CRT, defined as a relative decrease in ESV of ≥ 15%, was observed in nine patients (64%), and the remaining five patients (36%) were classified as non-responders. Compared with non-responders, responders were more likely to have higher ASDI (4.8 ± 1.1% vs. 2.8 ± 0.7%, p < 0.005) (Table 1). In contrast, there was no significant difference between responders and non-responders in the IVMD, Yu index, and 2-D radial dyssynchrony determined by speckle tracking strain (Table 1).

### Predictor of response to CRT and LV reverse remodeling

Of the individual parameters including ASDI and conventional dyssynchrony measures, ASDI ≥ 3.8% proved to be the best predictor of response to CRT with a sensitivity of 78%, specificity of 100% and area under the curve (AUC) of 0.93 (p < 0.001, Table 2). 2-D radial dyssynchrony was also found to be a successful predictors for response to CRT with an AUC of 0.82 (p < 0.005, Table 2). IVMD and Yu index, on the other hand, were not predictive of response to CRT.

**Table 2:** Relationship between individual parameters and response to cardiac resynchronization therapy

	AUC (95% CI)	Cut-off	Sensitivity (95% CI)	Specificity (95% CI)	p value
Area strain dyssynchrony index	0.93 (0.67-0.99)	3.8%	78% (40%-97%)	100% (48%-100%)	<0.001
Conventional dyssynchrony measurements					
IVMD	0.73 (0.44-0.93)	40 ms	67% (30%-93%)	60% (15%-95%)	NS
Yu Index	0.62 (0.33-0.86)	32 ms	11% (0.3%-48%)	100% (48%-100%)	NS
Radial dyssynchrony determined by speckle tracking strain	0.82 (0.53-0.97)	130 ms	100% (66%-100%)	60% (15%-95%)	<0.005

AUC, area under the receiver operating characteristic curve; CI, confidence interval; IVMD, interventricular mechanical delay.

Using pre-defined cut-off values for the conventional dyssynchrony parameters, sensitivity of IVMD, Yu index, and 2-D radial dyssynchrony was found to be 67%, 11%, and 100%, and specificity 60%, 100%, and 60%, respectively (Table 2).

**Figure 4:**
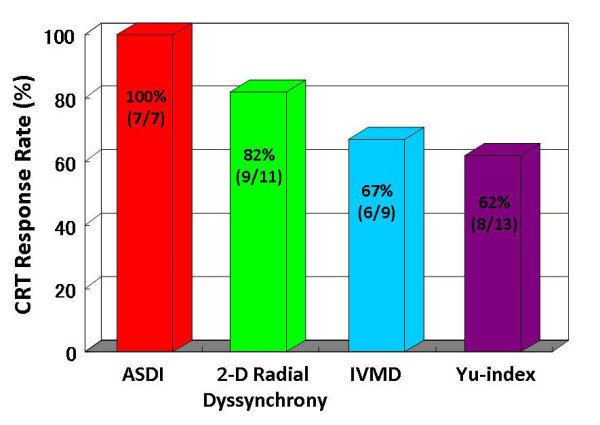
**Bar graphs showing comparison of rates of response to cardiac resynchronization therapy (CRT)**. Patients with strain dyssynchrony index with area tracking (ASDI) ≥ 3.8% showed a response rate of 100% (7/7), those with 2-D radial dyssynchrony determined by speckle tracking strain ≥ 130 msec a rate of 82% (9/11), those with interventricular mechanical delay (IVMD) 40 ≥ msec a rate of 67% (6/9), and those with a Yu index ≥ 32 msec a rate of 62% (8/13) CRT.

**Figure 5:**
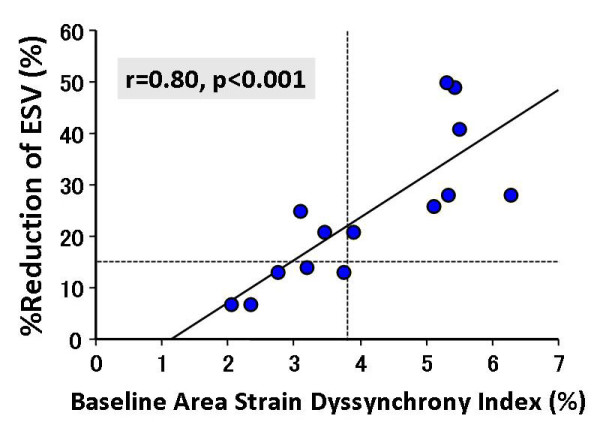
**Dot plot of % reductions in end-systolic volume (ESV) in relation to area strain dyssynchrony index shows a significant correlation**.

Noteworthy was that, ASDI ≥ 3.8% was associated with the highest incidence of clinical improvement after CRT with a response rate of 100% (7/7). On the other hand, pre-defined cut-off values of the conventional dyssynchrony parameters showed that CRT response rates were 67% (6/9), 62% (8/13), and 82% (9/11), respectively (Figure 4). Furthermore, baseline ASDI correlated significantly with % reduction in ESV after CRT (r = 0.80, p < 0.001; Figure 5). In contrast, there was no correlation between the conventional dyssynchrony parameters and % reduction in ESV. Figure 3 shows representative findings of baseline ASDI for a post-CRT responder patient.

### Reproducibility

The intraclass correlation coefficient for interobserver reproducibility was 0.961 (95% CI, 0.842 to 0.990), and the intraclass correlation coefficient for intraobserver reproducibility was 0.966 (95% CI, 0.864 to 0.992).

## Discussion

The findings of our study demonstrate that ASDI as determined by means of 3-D speckle area tracking imaging can predict response to CRT, and baseline ASDI may be capable of predicting LV reverse remodeling following CRT. This is the first study to assess the utility of novel 3-D speckle area tracking strain for predicting response to CRT and LV reverse remodeling.

### Importance of Combined Approach for Predicting Response to CRT

CRT is an established therapeutic option for HF patients with severe symptoms and a wide QRS complex[1-8]. Although initial results have been promising, roughly one-third of patients selected according to standard clinical criteria do not respond to CRT. Tissue Doppler imaging was useful to evaluate LV longitudinal function [24], and a number of publications to quantify LV dyssynchrony and predict response to CRT has focused on LV longitudinal shortening velocities on tissue Doppler imaging from the apical views. However, the ability of echocardiographic measures of dyssynchrony, in particular tissue Doppler imaging to predict response to CRT, has recently been criticized as a result of the findings by the predictors of responders to CRT (PROSPECT) trial[14]. On the contrary, speckle tracking strain has the advantage of differentiating active contraction from passive motion and is not affected by Doppler angle of incidence. Since response to CRT may be associated with LV mechanical dyssynchrony[5,6,17,25], LV lead position[17,26-29], scar burden or myocardial viability[30-34], irreversibly advanced HF[34], and AV and VV optimization, a comprehensive approach addressing these factors may well be required to minimize non-responders to CRT. The strain delay index reported by Lim et al[9] and our relatively simple version of the strain dyssynchrony index[10], both determined by 2-D speckle tracking strain, proved to be strong predictor of response to CRT. These two indexes combined thus constitute a marker of both LV mechanical dyssynchrony and residual myocardial contractility.

The LV myocardial function represents three different patterns of myocardial deformation, including radial direction (myocardial thickening), circumferential direction (myocardial shortening), and longitudinal direction (myocardial shortening). Because LV dyssynchrony is also a 3-D phenomenon, these three types of deformation do not provide the same information about the failing heart. STAR (the Speckle Tracking and Resynchronization) study, which is the recently conducted first prospective multi-centre study to assess the utility of radial, circumferential, transverse and longitudinal speckle tracking strain for predicting response to CRT and important long-term outcome events after CRT, verified the utility of multidirectional analysis for the quantification of LV dyssynchrony[25]. This study demonstrated that patients who lacked dyssynchrony before CRT, as determined by either the 2-D radial or transverse speckle tracking strain approach, suffered had serious unfavorable clinical events three times more frequently than those with significant baseline dyssynchrony. Furthermore, lack of dyssynchrony before CRT, as determined by the combined use of 2-D radial and transverse speckle tracking strains, was associated with implantation of a left ventricular assist device, heart transplant or death in approximately 50% of patients, in contrast to these unfavorable events occurring in 11-13% of patients if baseline 2-D radial or transverse speckle tracking dyssynchrony were present. Moreover, we previously reported that combining assessment of 2-D radial, circumferential, and longitudinal strain dyssynchrony indexes can enhance the prediction of CRT responders[10].

In this study, we found that baseline ASDI, as determined by means of novel 3-D speckle area tracking strain can predict response to CRT as well as predict LV reverse remodeling following CRT. 3-D area strain may provide new and more predictive endocardial information, because 3-D area strain coupled with both 3-D longitudinal and circumferential factors pertaining to the endocardium is considered to be the most sensitive to changes in myocardial function, especially in the failing heart or as a result of ischemia. 3-D area strain thus appears to be the ideal parameter to express LV dyssynchorny[35]. Furthermore, ASDI, which represents circumferential and longitudinal mechanical dyssynchrony and residual endomyocardial function, provide more comprehensive information for predicting response to CRT than do other methods.

3-D speckle tracking strain method was a novel developed technology and used in a few human studies. The potential advantages of 3-D speckle strain method were expression of myocardial/endocardial function of the whole heart, independency of tomographic imaging planes, and being able to analyze regional ventricular function using 3 different 3-D strains (radial/transverse, circumferential, and longitudinal) from the same heart beat acquisition. On the contrary, the potential disadvantages were relatively low volume rate of 25-30 volume/sec and relatively low spatial resolution. However, these disadvantages will be improved with technical development in the future.

### Clinical implications

3-D speckle tracking is a simple, feasible, and reproducible method for quantifying LV dyssynchrony[11,12], and it is considered to be faster than 2-D speckle tracking strain analysis[36]. As previously mentioned, comprehensive assessment may be crucial for selecting HF patients who will benefit most from CRT. It therefore appears that ASDI, which represents circumferential and longitudinal mechanical dyssynchrony as well as residual endomyocardial function, could be an alternative method for the assessment of HF patients in order to minimize non-responders to CRT. In view of the present study, the clinical algorithm is that a patient, whose ASDI is >3.8%, should be considered undergoing CRT.

### Study Limitations

Because the area tracking by means of 3-D speckle tracking system was developed only recently, this study covered a very small number of patients in a single-center study. Future studies of larger patient populations are therefore needed to test the accuracy of ASDI for predicting response to CRT and LV reverse remodeling. Moreover, the problem of the small number of the patients may be enhanced by the heterogeneous study population including 3 patients with ischemic cardiomyopathy and 4 patients with previously undergone implantation of a permanent RV pacing device. However, previous investigators have demonstrated that HF patients with RV pacing had similar dyssynchronous patterns and similar EF response and long-term outcome compared to those with left bundle branch block[11]. A limitation of the image acquisition for 3-D speckle tracking is the relatively slow volume rate of 25 to 30 volumes/s. The much slower volume rates of 3-D speckle tracking system compared to 2-D may limit analysis of rapid events such as isovolumic contraction and relaxation phase. Nevertheless, the reproducibility of ASDI in this study was acceptable. Finally, this 3-D speckle tracking strain methodology has been validated against sonomicrometry in animals[37], but there is no true non-invasive "gold standard" technique that can be used in humans to validate regional ventricular function. Therefore, this 3-D speckle tracking strain method does not establish the accuracy in humans.

## Conclusions

ASDI can predict responders and LV reverse remodeling following CRT. This novel index using the 3-D speckle area tracking system, which shows circumferential and longitudinal LV dyssynchrony and residual endocardial contractility, thus have clinical significance for CRT patients.

## List of abbreviations

CRT: cardiac resynchronization therapy; 3-D: three-dimensional; AV: atrioventricular; VV: venoventricular; LV: left ventricular; RV: right ventricular; ASDI: strain dyssynchrony index using area tracking; AUC: area under the curve; ESV: end-systolic volume; IVMD: interventricular mechanical delay.

## Competing interests

The authors declare that they have no competing interests.

## Authors' contributions

KT designed the study, carried out subject recruitment, performed echocardiography, analysed the data, and wrote the manuscript. AY performed biventricular pacemaker implantation. TT, AK, KY, KR, AO, YF, KN, KM, and TO assisted recruitment and manuscript revision. HT, HK, and KH assisted in study design, data interpretation and manuscript revision.

All authors read and approved the final manuscript.
